# Synthesis of phenanthridines via palladium-catalyzed picolinamide-directed sequential C–H functionalization

**DOI:** 10.3762/bjoc.9.102

**Published:** 2013-05-08

**Authors:** Ryan Pearson, Shuyu Zhang, Gang He, Nicola Edwards, Gong Chen

**Affiliations:** 1 Department of Chemistry, The Pennsylvania State University, University Park, Pennsylvania 16802, United States of America; 2 Department of Chemistry, The Pennsylvania State University, Worthington Scranton, Dunmore, Pennsylvania 18512, United States of America

**Keywords:** C–H functionalization, palladium, phenanthridine, picolinamide

## Abstract

We report a new synthesis of phenanthridines based on palladium-catalyzed picolinamide-directed sequential C–H functionalization reactions starting from readily available benzylamine and aryl iodide precursors. Under the catalysis of Pd(OAc)_2_, the *ortho*-C–H bond of benzylpicolinamides is first arylated with an aryl iodide. The resulting biaryl compound is then subjected to palladium-catalyzed picolinamide-directed intramolecular dehydrogenative C–H amination with PhI(OAc)_2_ oxidant to form the corresponding cyclized dihydrophenanthridines. The benzylic position of these dihydrophenanthridines could be further oxidized with Cu(OAc)_2_, removing the picolinamide group and providing phenathridine products. The cyclization and oxidation could be carried out in a single step and afford phenathridines in moderate to good yields.

## Introduction

**Scheme 1:**
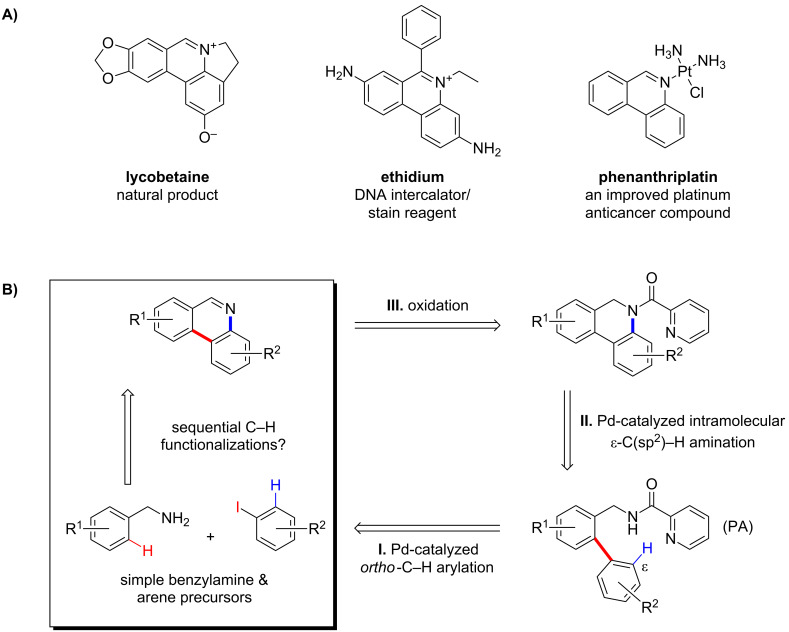
Representative phenanthridine compounds and our synthetic strategy based on Pd-catalyzed sequential C–H functionalizations.

Phenanthridines and 5,6-dihydro-phenanthridines are important core structures found in a variety of natural products and functional molecules (Scheme 1A) [1–8]. Synthetic methods for their preparation include the classical Pictet–Hubert condensation [9], radical-mediated reactions [10–13], metal-catalyzed cross-couplings [14–18], cycloadditions [19], and others [20–22]. More recently, methods based on the metal-catalyzed functionalization of carbon–hydrogen (C–H) bonds have also emerged as viable strategies for synthesizing phenanthridines [23–25]. Despite these advances, construction of phenanthridines with complex substitution patterns remains difficult and often requires lengthy and inefficient synthetic sequences. Herein, we report a novel method for phenanthridine synthesis based on sequential palladium-catalyzed picolinamide (PA)-directed C–H functionalization reactions beginning from easily accessible PA-protected benzylamine and aryl iodide precursors.

## Results and Discussion

**New synthetic strategy for phenanthridine compounds.** The picolinamide (PA) group has been shown to be an excellent directing group for a range of Pd-catalyzed C–H functionalization reactions [26–35]. In 2005, the Daugulis laboratory first reported that the *ortho*-C(sp^2^)−H bond of benzylpicolinamides could be arylated with aryl iodides under Ag-promoted Pd-catalyzed conditions [26]. In 2012, our laboratory [28] as well as that of Daugulis [27] independently reported that picolinamide substrates can undergo intramolecular dehydrogenative C–H amination reactions to afford medium-sized *N*-heterocycles under the catalysis of Pd(OAc)_2_ with PhI(OAc)_2_ oxidant. These discoveries led us to explore whether we could develop a new strategy for synthesizing phenanthridines. As outlined in Scheme 1B, we envisioned that *ortho*-arylated benzylamine picolinamides could undergo an intramolecular amination at the *ortho* ε-C–H position of the newly installed arene group to form cyclized dihydrophenanthridines, which could be further converted to phenanthridine products under oxidative conditions. Ideally, we hoped to perform both the intramolecular C–H amination and subsequent oxidation in a single step [36].

**Arylation of 2-methoxybenzyl picolinamide 1 with 4-iodoanisole (2) under various conditions**. We commenced the study by investigating the arylation of 2-methoxybenzyl picolinamide **1** with 4-iodoanisole (**2**) under various conditions (Table 1) to form our desired arylated product **3**. Our initial attempt under the original Pd(OAc)_2_-catalyzed AgOAc-promoted solvent-free condition afforded the desired arylated product **3** in good yield (Table 1, entry 1). This method, however, required the use of expensive silver salt as an additive and high reaction temperature (150 °C). We next sought to replace the silver salts with cheaper reagents and lower the reaction temperature [12]. Not surprisingly, the arylation yield dropped significantly when the reaction was performed in toluene solvent at 120 °C (Table 1, entry 2). Addition of PivOH (0.3 equiv) gave little improvement (Table 1, entries 3 and 4). To our delight, the desired arylation reaction was largely restored with the application of 2 equiv of K_2_CO_3_ at 120 °C for 24 h (Table 1, entry 5). Furthermore, an excellent yield was obtained when K_2_CO_3_ was replaced with KHCO_3_ and 0.3 equiv of PivOH was applied (Table 1, entry 7). The most effective carboxylate ligand and solvent was found to be PivOH and toluene, respectively.

**Table 1:** Optimization of the Pd-catalyzed *ortho*-C–H arylation of benzylpicolinamide. All screening reactions were carried out in a 10 mL glass vial on a 0.2 mmol scale.

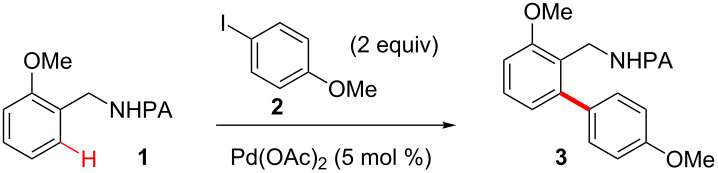				

entry	additives (equiv)	temperature (°C)	solvent	yield of **3** (%)^a^

1	AgOAc (1.5)	150	no solvent	76
2	AgOAc (1.5)	120	toluene	6
3	AgOAc (1.5), PivOH (0.3)	120	toluene	3
4	PivOH (0.3)	120	toluene	5
5	K_2_CO_3_ (2)	120	toluene	57
6	PivOH (0.3), K_2_CO_3_ (2)	120	toluene	90
7	PivOH (0.3), KHCO_3_ (2)	120	toluene	95 (91)^b^
8	AcOH (0.3), KHCO_3_ (2)	120	toluene	78
9	*o*PBA^c^ (0.3), KHCO_3_ (2)	120	toluene	84
10	PivOH (0.3), KHCO_3_ (2)	90	toluene	29

^a^Yields are based on ^1^H NMR analysis of the reaction mixture after workup; ^b^Isolated yield; ^c^*o*PBA: *ortho*-phenylbenzoic acid.

**Figure 1:**
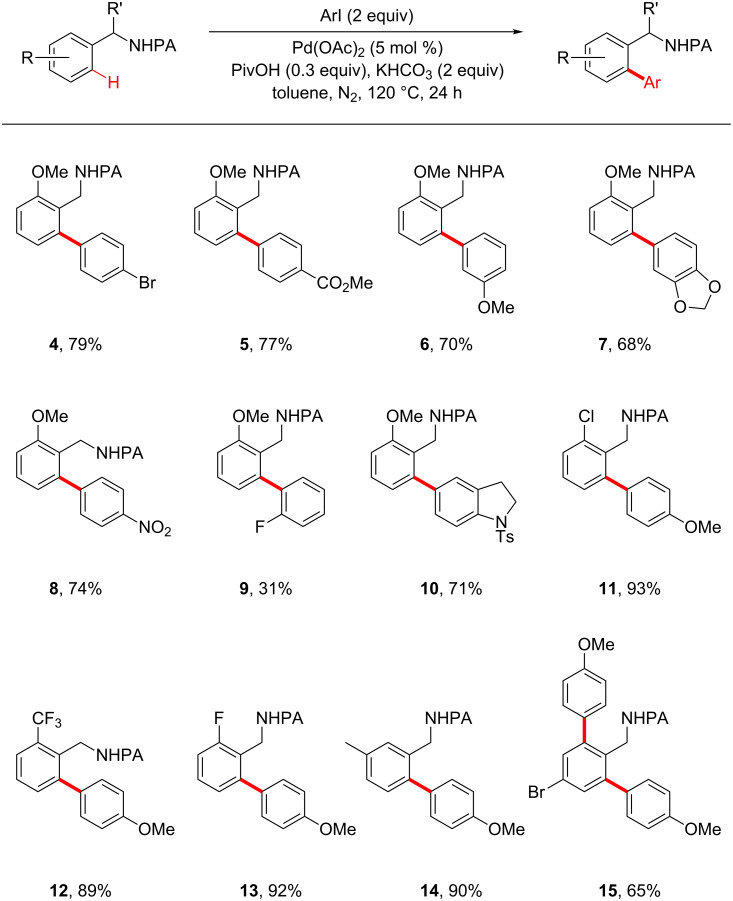
Substrate scope of the Pd-catalyzed PA-directed C–H arylation reaction. All reactions were carried out in a 10 mL glass vial on a 0.2 mmol scale; yields are based on the isolated product.

**The determination of the scope of this reaction with benzylpicolinamide and aryl iodide substrates.** With the optimized conditions in hand, we next explored the scope of benzylpicolinamide and aryl iodide substrates (Figure 1). The electronic properties of benzylpicolinamide and aryl iodides had little influence on the reactivity, as benzylpicolinamide and aryl iodide substrates bearing electron-donating and withdrawing substituents react in good yields (**3**, **8**, and **12**). Significantly decreased arylation yield was observed for *ortho*-substituted aryl iodides (e.g., **9**). The sterics of the benzylpicolinamides is also important for the regioselectivity of the arylation reaction. For instance, the less hindered *ortho* position is preferentially arylated (e.g., **14**) when a *meta* substitutent is present on the benzylpicolinamide. Aryl bromides are much less reactive compared with aryl iodide substrates **4**. This is in accordance with results on the Pd-catalyzed PA-directed arylation of more inert C(sp^3^)−H bonds [29].

**Cyclization of biaryl compounds to form dihydrophenanthridines**. Next, we investigated the cyclization of biaryl compounds to form dihydrophenanthridines via Pd-catalyzed intramolecular dehydrogenative amination of ε-C(sp^2^)–H bonds [37–45]. To our delight, treatment of **3** in the presence of 5 mol % of Pd(OAc)_2_ and 2 equiv of PhI(OAc)_2_ in toluene at 120 °C for 24 h gave the desired dihydrophenanthridine **16** in good yield (Table 2, entry 1). In addition, a further oxidized phenanthridine **17** was obtained as a side product. Compound **17** is presumably generated through the PhI(OAc)_2_-mediated oxidation of the benzylic C–H bond to form a phenanthridinium intermediate **18**, which then undergoes a removal of the PA group. Encouraged by these observations, we proceeded to explore whether the cyclization and oxidation steps can be performed in one step to give the phenanthridines in a shorter procedure. A variety of oxidants, such as 1,4-benzoquinone (BQ), KMnO_4_, ceric ammonium nitrate (CAN), and copper salts were examined [46]. The combination of PhI(OAc)_2_ (2 equiv) and Cu(OAc)_2_ (2 equiv) afforded the phenanthridine product **17** in highest yield (Table 2, entry 7). The yield can be further improved using 10 mol % of Pd(OAc)_2_ catalyst (Table 2, entry 8). In a control experiment, dihydrophenanthridine **16** was oxidized with the application of Cu(OAc)_2_ (2 equiv) in toluene at 120 °C for 24 h, forming **17** in excellent yield. We believe that PhI(OAc)_2_ serves as the oxidant for the initial Pd-catalyzed intramolecular C–H amination step, in which a Pd^II/IV^ catalytic manifold might be operative. Cu(OAc)_2_ is responsible for the subsequent oxidation of the benzylic C–H bond of dihydrophenanthridine.

**Table 2:** Formation of phenanthridine **17** in a single step by Pd-catalyzed intramolecular C–H amination followed by oxidation. All screening reactions were carried out in a 10 mL glass vial on a 0.2 mmol scale.

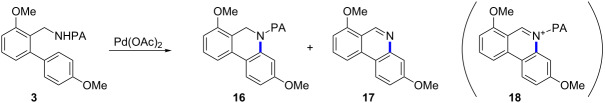				

entry	Pd(OAc)_2_ (mol %)	additives (equiv)	yield (%)^a^	
			**16**	**17**

1	5	PhI(OAc)_2_ (2)	40	5
2	5	PhI(OAc)_2_ (2), AcOH (2)	23	8
3	5	PhI(OAc)_2_ (2), BQ (2)	35	10
4	5	PhI(OAc)_2_ (2), KMnO_4_ (2)	56	3
5	5	PhI(OAc)_2_ (2), CAN (2)	37	25
6	5	PhI(OAc)_2_ (2), CuCl_2_ (2)	29	34
7	5	PhI(OAc)_2_ (2), Cu(OAc)_2_ (2)	17	51
8	10	PhI(OAc)_2_ (2), Cu(OAc)_2_ (2)	15	62 (58)^b^

^a^Yields are based on ^1^H NMR analysis of the reaction mixture after workup; ^b^Isolated yield.

**Figure 2:**
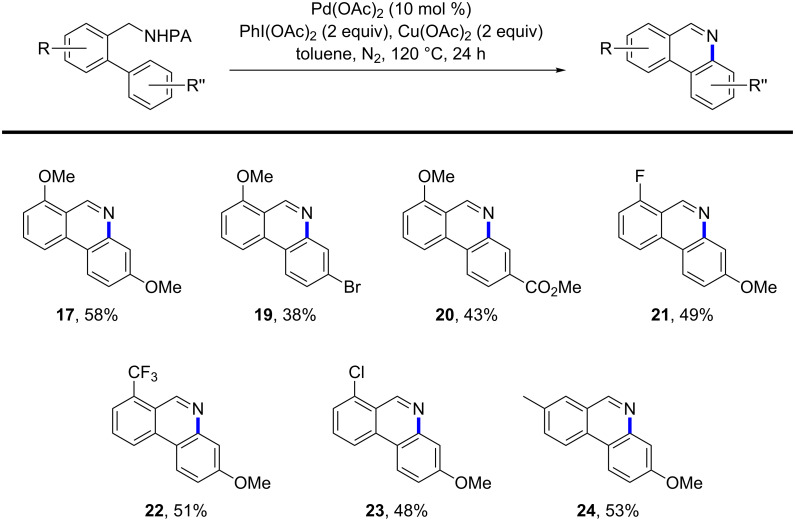
Substrate scope of this phenanthridine synthesis. All reactions were carried out in a 10 mL glass vial on a 0.2 mmol scale; yields are based on isolated product.

**Extension of the cyclization–oxidation step to other arylated picolinamide substrates.** The coupled cyclization–oxidation step detailed above was then used to synthesize phenanthridines from other arylated picolinamide substrates (Figure 2). In general, electron-rich arene motifs, installed by C–H arylation, gave a higher yield of phenanthridine products; electron-deficient substrates provide a lower yield. For instance, substrate **8** with a *para*-nitro group failed to give any cyclized product under the standard conditions. Substrates with moderately electron-withdrawing groups, such as **20** bearing a *para-*ester group, reacted in moderate yield. The electronic properties of the benzylpicolinamide scaffold had much less influence on the reaction. For example, product **22** bearing an *ortho*-CF_3_ substituent was obtained in 51% yield. Finally, it is noteworthy that all of the above phenanthridine products show intense blue fluorescence. We expect our synthetic strategy will afford access to phenanthridines bearing varied substitution patterns, enabling applications in biology and materials science.

## Conclusion

In summary, we have developed a readily applicable two-step method for the synthesis of phenanthridines from easily accessible benzylamine picolinamides and aryl iodides. In the first step, an improved protocol allows us to carry out the Pd-catalyzed PA-directed C–H arylation reaction without the use of expensive silver additives. In the second step, application of PhI(OAc)_2_ and Cu(OAc)_2_ oxidant under the catalysis of Pd(OAc)_2_ affords phenanthridines in moderate to good yields. Applications of this method to the synthesis of more complex phenanthridines with novel photophysical properties are currently underway.

## Experimental

**General conditions:** All commercial materials were used as received unless otherwise noted. All solvents were obtained from a JC Meyer solvent dispensing system and used without further purification. Flash chromatography was performed using 230–400 mesh SiliaFlash 60^®^ silica gel (Silicycle Inc.). PhI(OAc)_2_ (98%, Aldrich), Pd(OAc)_2_ (98%, Aldrich) were used in the Pd-catalyzed reactions. NMR spectra were recorded on Bruker CDPX-300, DPX-300, DPX-400 instruments and calibrated by using residual solvent peaks as the internal reference. Multiplicities are recorded as: s = singlet, d = doublet, t = triplet, dd = doublet of doublets, m = multiplet. High-resolution ESI mass experiments were operated on a Waters LCT Premier instrument.

**Standard procedure for the Pd-catalyzed *ortho* C–H arylation reaction:** A mixture of picolinamide **1** [30] (48 mg, 0.2 mmol, 1 equiv), aryl iodide **2** (94 mg, 0.4 mmol, 2 equiv), Pd(OAc)_2_ (2.2 mg, 0.01 mmol, 0.05 equiv), KHCO_3_ (40 mg, 0.4 mmol, 2.0 equiv), and PivOH (6 mg, 0.06 mmol, 0.3 equiv) in anhydrous toluene (4 mL) in a 10 mL glass vial (purged with N_2_, sealed with PTFE cap) was heated at 120 °C for 24 h. The reaction mixture was filtered through a short pad of celite and concentrated in vacuo. The resulting residue was purified by silica-gel flash chromatography (hexanes/EtOAc 3:1) to give the product **3** as a pale white solid (64 mg, 91%). Compounds **4**–**15** were prepared from the known precursors [30] by using the standard C–H arylation procedure.

**Standard procedure for the Pd-catalyzed cyclization and oxidation reaction to form phenanthridines:** A mixture of picolinamide **3** (70 mg, 0.2 mmol, 1 equiv), Pd(OAc)_2_ (4.4 mg, 0.02 mmol, 0.1 equiv), PhI(OAc)_2_ (129 mg, 0.4 mmol, 2.0 equiv), and Cu(OAc)_2_ (72 mg, 0.4 mmol, 2 equiv) in anhydrous toluene (4 mL) in a 10 mL glass vial (purged with N_2_, sealed with PTFE cap) was heated at 120 °C for 24 h. The reaction mixture was filtered through a short pad of celite and concentrated in vacuo. The resulting residue was purified by silica gel flash chromatography (hexanes/EtOAc 4:1) to give the product **17** as a pale white solid (28 mg, 58%). Compounds **19**–**24** were prepared by using the standard cyclization–oxidation procedure.

**Compound 3**. ^1^H NMR (CDCl_3_, 300 MHz) δ 8.53 (d, *J* = 4.2 Hz, 1H), 8.40 (s, 1H), 8.21 (d, *J* = 7.5 Hz, 1H), 7.85–7.80 (m, 1H), 7.42–7.30 (m, 3H), 6.99–6.93 (m, 4H), 4.65 (d, *J* = 5.4 Hz, 2H), 3.95 (s, 3H), 3.85 (s, 3H); ^13^C NMR (CDCl_3_, 75 MHz) δ 163.3, 158.8, 158.6, 150.2, 147.9, 143.6, 137.1, 132.7, 130.3, 128.3, 125.8, 123.4, 122.7, 122.1, 113.6, 109.1, 55.7, 55.2, 36.8; HRMS (*m*/*z*): [M + H]^+^ calcd for C_21_H_21_N_2_O_3_, 349.1552; found, 349.1546.

**Compound 4**. ^1^H NMR (CDCl_3_, 300 MHz) δ 8.52 (d, *J* = 4.5 Hz, 1H), 8.36 (s, 1H), 8.19 (d, *J* = 7.8 Hz, 1H), 7.84 (td, *J* = 7.8, 1.4 Hz, 1H), 7.55 (d, *J* = 8.3 Hz, 2H), 7.41 (dd, *J* = 5.0, 6.8 Hz, 1H), 7.35 (t, *J* = 8.0 Hz, 1H), 7.25 (d, *J* = 8.3 Hz, 2H), 6.97 (d, *J* = 8.2 Hz, 1H), 6.89 (d, *J* = 7.7 Hz, 1H), 4.59 (d, *J* = 5.5 Hz, 2H), 3.95 (s, 3H); ^13^C NMR (CDCl_3_, 75.5 MHz) δ 163.3, 158.7, 150.1, 148.0, 142.5, 139.4, 137.1, 131.3, 130.9, 128.6, 125.9, 123.5, 122.4, 122.1, 121.6, 109.9, 55.8, 36.7; HRMS (*m*/*z*): [M + H]^+^ calcd for C_20_H_18_BrN_2_O_2_, 397.0552; found, 397.0561.

**Compound 5**. ^1^H NMR (CDCl_3_, 400 MHz) δ 8.51 (d, *J* = 4.0 Hz, 1H), 8.33 (s, 1H), 8.15 (d, *J* = 7.6 Hz, 1H), 8.08 (d, *J* = 8.0 Hz, 1H), 7.80 (t, *J* = 7.6 Hz, 1H), 7.44–7.34 (m, 4H), 6.97 (d, *J* = 8.0 Hz, 1H), 6.90 (d, *J* = 7.6 Hz, 1H), 4.58 (d, *J* = 5.2 Hz, 2H), 3.95 (s, 3H), 3.92 (s, 3H); ^13^C NMR (CDCl_3_, 75 MHz) δ 166.9, 163.3, 158.7, 150.2, 148.0, 145.2, 142.9, 137.2, 129.5, 129.3, 129.0, 128.6, 125.9, 123.5, 122.3, 122.2, 110.0, 55.8, 52.1, 36.6; HRMS (*m*/*z*): [M + H]^+^ calcd for C_22_H_21_N_2_O_4_, 377.1501; found, 377.1509.

**Compound 6**. ^1^H NMR (CDCl_3_, 300 MHz) δ 8.51 (d, *J* = 4.2 Hz, 1H), 8.34 (s, 1H), 8.19 (d, *J* = 7.8 Hz, 1H), 7.40–7.31 (m, 1H), 6.98–6.91 (m, 5H), 4.66 (d, *J* = 5.4 Hz, 2H), 3.94 (s, 3H), 3.79 (s, 3H); ^13^C NMR (CDCl_3_, 75 MHz) δ 163.8, 159.7, 159.0, 150.7, 148.4, 144.4, 142.3, 137.6, 129.7, 128.9, 126.3, 124.0, 123.0, 122.6, 122.1, 114.9, 113.8, 110.1, 55.3, 55.6, 37.2; HRMS (*m*/*z*): [M + H]^+^ calcd for C_21_H_21_N_2_O_3_, 349.1552; found, 349.1564.

**Compound 7**. ^1^H NMR (CDCl_3_, 300 MHz) δ 8.53 (d, *J* = 4.2 Hz, 1H), 8.34 (s, 1H), 8.19 (d, *J* = 7.8 Hz, 1H), 7.84–7.80 (m, 1H), 7.40–7.29 (m, 2H), 6.96–6.80 (m, 5H), 6.00 (s, 2H), 4.64 (d, *J* = 5.4 Hz, 1H), 3.96 (s, 3H); ^13^C NMR (CDCl_3_, 75 MHz) δ 163.4, 158.7, 150.3, 148.0, 147.4, 146.9, 143.7, 137.1, 134.4, 128.4, 125.8, 123.7, 122.8, 122.7, 122.2, 109.9, 109.5, 108.1, 101.0, 55.8, 36.8; HRMS (*m*/*z*): [M + H]^+^ calcd for C_21_H_20_N_2_O_4_, 363.1345; found, 363.1355.

**Compound 8**. ^1^H NMR (CDCl_3_, 300 MHz) δ 8.53 (d, *J* = 4.1 Hz, 1H), 8.41 (s, 1H), 8.29 (d, *J* = 8.8 Hz, 2H), 8.16 (d, *J* = 7.8 Hz, 1H), 7.85 (td, *J* = 7.7, 1.6 Hz, 1H), 7.55 (d, *J* = 8.7 Hz, 2H), 7.42–7.34 (m, 2H), 7.03 (d, *J* = 8.2 Hz, 1H), 6.89 (d, *J* = 7.7 Hz, 1H), 4.58 (d, *J* = 5.7 Hz, 2H), 3.98 (s, 3H); ^13^C NMR (CDCl_3_, 75.5 MHz) δ 163.4, 158.8, 150.0, 148.1, 147.4, 147.1, 141.4, 137.3, 130.4, 128.9, 126.1, 123.7, 123.5, 122.2, 122.2, 110.7, 56.0, 36.6; HRMS (*m*/*z*): [M + H]^+^ calcd for C_20_H_18_N_3_O_4_, 364.1297; found, 364.1292.

**Compound 9**. ^1^H NMR (CDCl_3_, 300 MHz) δ 8.53 (d, *J* = 4.2 Hz, 1H), 8.32 (s, 1H), 8.18–8.16 (m, 1H), 7.85–7.80 (m, 1H), 7.38–7.30 (m, 4H), 7.25–7.13 (m, 2H), 7.02–6.92 (m, 2H), 4.64–4.90 (m, 2H), 3.98 (s, 3H); ^13^C NMR (CDCl_3_, 75 MHz) δ 163.8, 161.2, 158.9, 150.7, 148.4, 137.8, 137.6, 132.0, 129.9, 128.9, 126.2, 125.2, 124.5, 123.3, 122.6, 115.9, 115.8, 110.7, 56.22, 37.2; HRMS (*m*/*z*): [M + H]^+^ calcd for C_20_H_18_FN_2_O_2_, 337.1352; found, 337.1364.

**Compound 10**. ^1^H NMR (CDCl_3_, 300 MHz) δ 8.51 (d, *J* = 4.0 Hz, 1H), 8.25 (s, 1H), 8.17 (d, *J* = 7.8 Hz, 1H), 7.84 (td, *J* = 7.7, 1.7 Hz, 1H), 7.73 (d, *J* = 8.3 Hz, 2H), 7.66 (d, *J* = 8.3 Hz, 1H), 7.41–7.36 (m, 1H), 7.32–7.24 (m, 3H), 7.17 (d, *J* = 8.3 Hz, 1H), 7.06 (s, 1H), 6.94 (d, *J* = 8.2 Hz, 1H), 6.88 (d, *J* = 7.4 Hz, 1H), 4.57 (d, *J* = 5.4 Hz, 2H), 3.95 (t, *J* = 8.8 Hz, 2H), 3.92 (s, 3H), 2.93 (t, *J* = 8.4 Hz, 2H), 2.37 (s, 3H); ^13^C NMR (CDCl_3_, 75.5 MHz) δ 163.3, 158.7, 150.2, 148.1, 144.1, 143.4, 141.3, 137.2, 136.1, 133.9, 131.8, 129.8, 128.7, 128.5, 127.3, 126.2, 125.9, 123.5, 122.6, 122.1, 114.5, 109.5, 55.8, 50.1, 36.7, 27.8, 21.5; HRMS (*m*/*z*): [M + H]^+^ calcd for C_29_H_18_N_3_O_4_S, 514.1801; found: 514.1813.

**Compound 11**. ^1^H NMR (CDCl_3_, 300 MHz) δ 8.54 (d, *J* = 3.9 Hz, 1H), 8.21 (d, *J* = 7.5 Hz, 2H), 7.88–7.83 (m, 1H), 7.46–7.41 (m, 2 H), 7.34–7.23 (m, 4 H), 6.96 (d, *J* = 8.7 Hz, 1H), 4.72 (d, *J* = 5.4 Hz, 1H), 8.86 (s, 3H); ^13^C NMR (CDCl_3_, 75 MHz) δ 163.5, 159.1, 149.8, 148.0, 144.9, 137.2, 135.8, 132.9, 132.4, 130.1, 129.2, 128.7, 128.6, 126.0, 122.2, 113.8, 55.2, 39.6; HRMS (*m*/*z*): [M + H]^+^ calcd for C_20_H_18_ClN_2_O_2_, 353.1057; found, 353.1067.

**Compound 12**. ^1^H NMR (CDCl_3_, 300 MHz) δ 8.50 (d, *J* = 4.8 Hz, 1H), 8.14 (d, *J* = 7.8 Hz, 1H), 7.93 (s, 1H), 7.83–7.74 (m, 2H), 7.50–7.39 (m, 3H), 7.28–7.22 (m, 2H), 6.89 (d, *J* = 8.7 Hz, 2H), 4.71 (d, *J* = 4.5 Hz, 1H), 3.82 (s, 3H); ^13^C NMR (CDCl_3_, 75 MHz) δ 163.0, 159.1, 149.6, 148.0, 145.6, 137.1, 134.6, 133.5, 131.9, 130.1, 129.9, 127.8, 126.0, 125.4, 122.1, 113.8, 55.2, 38.2; HRMS (*m*/*z*): [M + H]^+^ calcd for C_21_H_18_F_3_N_2_O_2_, 387.1320; found, 387.1328.

**Compound 13**. ^1^H NMR (CDCl_3_, 300 MHz) δ 8.51 (d, *J* = 4.2 Hz, 1H), 8.18 (d, *J* = 7.8 Hz, 2H), 7.83–7.79 (m, 1H), 7.42–7.38 (m, 1H), 7.33–7.30 (m, 3H), 7.11 (d, *J* = 7.5 Hz, 2H), 6.97 (d, *J* = 8.7 Hz, 2H), 4.68 (d, *J* = 5.4 Hz, 2H), 3.84 (s, 3H); ^13^C NMR (CDCl_3_, 75 MHz) δ 163.7, 160.4, 159.1, 149.7, 147.9, 144.4, 137.1, 131.6, 130.1, 128.9, 128.7, 126.0, 122.6, 122.1, 114.3, 113.8, 55.2, 35.6; HRMS (*m*/*z*): [M + H]^+^ calcd for C_20_H_18_FN_2_O_2_, 337.1352; found, 337.1357.

**Compound 14**. ^1^H NMR (CDCl_3_, 300 MHz) δ 8.50 (d, *J* = 4.5 Hz, 1H), 8.27–8.21 (m, 2H), 7.82 (t, *J* = 7.5 Hz, 1H), 7.41–7.29 (m, 4H), 7.19 (dd, *J* = 14.1 and 7.8 Hz, 2H), 6.98 (d, *J* = 8.4 Hz, 2H), 4.64 (d, *J* = 6.0 Hz, 2H), 3.84 (s, 3H), 2.38 (s, 3H); ^13^C NMR (CDCl_3_, 75 MHz) δ 163.8, 158.5, 149.6, 147.8, 138.4, 137.0, 136.9, 135.2, 132.8, 130.0, 129.1, 127.9, 125.9, 122.0, 113.5, 55.0, 41.1, 20.9; HRMS (*m*/*z*): [M + H]^+^ calcd for C_21_H_21_N_2_O_2_, 333.1603; found, 333.1609.

**Compound 15**. ^1^H NMR (CDCl_3_, 300 MHz) δ 8.45 (d, *J* = 4.7 Hz, 1H), 8.01 (d, *J* = 7.8 Hz, 1H), 7.78 (m, 2H), 7.42 (s, 2H), 7.37 (m, 1H), 7.30 (d, *J* = 8.7 Hz, 4H), 6.90 (d, *J* = 8.7 Hz, 4H), 4.47 (d, *J* = 5.1 Hz, 2H), 3.79 (s, 6H); ^13^C NMR (CDCl_3_, 75.5 MHz) δ 162.9, 159.1, 149.5, 147.8, 145.2, 137.0, 132.3, 132.2, 132.1, 129.9, 125.9, 121.8, 121.0, 113.8, 55.2, 39.0; HRMS (*m*/*z*): [M + H]^+^ calcd for C_27_H_24_Br_3_N_2_O_3_, 503.0970; found, 503.0975.

**Compound 17**. ^1^H NMR (CDCl_3_, 300 MHz) δ 9.72 (s, 1H), 8.45 (d, *J* = 9.0 Hz, 1H), 8.08 (d, *J* = 8.2 Hz, 1H), 7.75 (t, *J* = 8.1 Hz, 1H), 7.61 (d, *J* = 2.6 Hz, 1H), 7.33–7.29 (m, 1H), 6.99 (d, *J* = 7.8 Hz, 1H), 4.09 (s, 3H), 4.00 (s, 3H); ^13^C NMR (CDCl_3_, 75.5 MHz) δ 160.5, 157.5, 149.1, 147.2, 134.6, 132.1, 124.3, 118.5, 117.1, 113.8, 110.1, 105.9, 56.2, 56.0; HRMS (*m*/*z*): [M + H]^+^ calcd for C_15_H_14_NO_2_, 240.1025; found, 240.1030.

**Compound 19**. ^1^H NMR (CDCl_3_, 400 MHz) δ 9.68 (s, 1H), 8.33 (m, 2H), 8.04 (d, *J* = 8.3 Hz, 1H), 7.75 (m, 2H), 7.04 (d, *J* = 7.9 Hz, 1H), 4.05 (s, 3H); ^13^C NMR (CDCl_3_, 75.5 MHz) δ 157.6, 149.5, 145.7, 133.8, 132.5, 132.2, 130.1, 124.3, 122.5, 117.4, 113.7, 107.2, 55.9; HRMS (*m*/*z*): [M + H]^+^ calcd for C_14_H_11_BrNO, 228.0024; found, 228.0032.

**Compound 20**. ^1^H NMR (CDCl_3_, 300 MHz) δ 9.75 (s, 1H), 8.84 (s, 1H), 8.54 (d, *J* = 8.7 Hz, 1H), 8.24 (d, *J* = 7.5 Hz, 1H), 8.14 (d, *J* = 8.4 Hz, 1H), 7.79 (d, *J* = 8.1 Hz, 1H), 7.09 (d, *J* = 8.1 Hz, 1H), 4.06 (s, 3H), 4.01 (s, 3H); ^13^C NMR (CDCl_3_, 75 MHz) δ 166.8, 157.4, 149.2, 144.0, 133.2, 132.1, 132.0, 130.1, 127.0, 126.7, 122.9, 117.9, 114.2, 107.8, 55.8, 52.4; HRMS (*m*/*z*): [M + H]^+^ calcd for C_16_H_13_NO_3_, 268.0974, found, 268.0970.

**Compound 21**. ^1^H NMR (CDCl_3_, 300 MHz) δ 9.59 (s, 1H), 8.46 (d, *J* = 9.0 Hz, 1H), 8.29 (d, *J* = 8.4 Hz, 1H), 7.82–7.74 (m, 1H), 7.64 (s, 1H), 7.37–7.26 (m, 2H), 4.01 (s, 3H); ^13^C NMR (CDCl_3_, 75 MHz) δ 160.6, 147.4, 146.6, 134.6, 131.7, 131.6, 123.8, 118.7, 117.4, 117.3, 111.1, 110.8, 110.1, 55.7; HRMS (*m*/*z*): [M + H]^+^ calcd for C_14_H_10_FNO, 228.0825; found, 228.0830.

**Compound 22**. ^1^H NMR (CDCl_3_, 300 MHz) δ 9.62 (s, 1H), 8.72 (d, *J* = 8.4 Hz, 1H), 8.47 (d, *J* = 9.0 Hz, 1H), 7.95 (d, *J* = 7.2 Hz, 1H), 7.85 (t, *J* = 8.1 Hz, 1H), 7.62 (d, *J* = 2.4 Hz, 1H), 7.36 (dd, *J* = 9.0 Hz and 2.7 Hz, 1H), 4.01 (s, 3H); ^13^C NMR (CDCl_3_, 75 MHz) δ 160.7, 149.3, 145.8, 133.8, 129.5, 125.8, 124.4, 124.3, 123.4, 122.2, 119.1, 117.6, 109.8, 55.6; HRMS (*m*/*z*): [M + H]^+^ calcd for C_15_H_12_F_3_NO, 278.0793; found, 278.0797.

**Compound 23**. ^1^H NMR (CDCl_3_, 300 MHz) δ 9.72 (s, 1H), 8.46–8.42 (m, 2H), 7.72–7.62 (m, 3H), 7.35 (dd, *J* = 9.0 and 2.4 Hz, 1H), 4.02 (s, 3H); ^13^C NMR (CDCl_3_, 75 MHz, ppm) δ 160.5, 150.1, 146.1, 134.5, 133.9, 131.0, 126.9, 123.6, 122.3, 120.4, 118.8, 117.1, 109.9, 55.6; HRMS (*m*/*z*): [M + H]^+^ calcd for C_14_H_11_ClNO, 244.0529; found, 244.0534.

**Compound 24**. ^1^H NMR (CDCl_3_, 300 MHz) δ 9.23 (s, 1H), 8.48–8.42 (m, 2H), 7.82 (s, 1H), 7.69 (d, *J* = 8.1 Hz, 1H), 7.62 (s, 1H), 7.34–7.30 (m, 1H), 4.02 (s, 3H), 2.62 (s, 3H); ^13^C NMR (CDCl_3_, 75 MHz) δ 160.2, 154.2, 146.3, 137.4, 136.7, 133.4, 131.1, 128.5, 123.6, 121.7, 118.2, 118.4, 110.3, 56.0, 21.9; HRMS (*m*/*z*): [M + H]^+^ calcd for C_15_H_13_NO, 244.1075; found, 244.1079.
